# Retrospective 3D analysis of gingival recession and occlusal morphology based on digital intraoral scans: an exploratory study

**DOI:** 10.1007/s00784-026-07204-z

**Published:** 2026-10-09

**Authors:** Tannaz Milani, Thomas Holzinger, Markus Laky, Xiaohui Rausch-Fan

**Affiliations:** 1 Clinical Division of Periodontology, University Clinic of Dentistry, Medical University of Vienna, Vienna, 1090 Austria; 2 Center for Clinical Research, University Clinic of Dentistry, Medical University of Vienna, Vienna, 1090 Austria; 3 Clinical Division of Conservative Dentistry, University Clinic of Dentistry, Medical University of Vienna, Vienna, 1090 Austria; 4 Competence Center Artificial Intelligence in Dentistry, University Clinic of Dentistry, Medical University of Vienna, Vienna, 1090 Austria; 5 Comprehensive Center for Artificial Intelligence in Medicine, Medical University of Vienna, Vienna, 1090 Austria

**Keywords:** Gingival recession, Intraoral scans, Occlusal wear, Crown angulation, Tooth alignment, Retrospective study

## Abstract

**Objectives:**

To characterize gingival recession (GR) in archived intraoral scans and to investigate associations with scan-visible occlusal and morphological features at patient and tooth levels.

**Materials and methods:**

This retrospective exploratory study included digital scans from adult patients without documented previous orthodontic treatment or relevant periodontal destruction. Mid-buccal GR was recorded as an ordinal score and as continuous depth. Wear-facet severity, a derived wear-facet involvement score, tooth type, alignment, and crown angulation were assessed. Patient-level associations were examined using logistic regression. Among recession-affected teeth, associations with recession severity were evaluated using cumulative link and gamma generalized linear mixed models accounting for clustering within patients. Exploratory random-forest modelling assessed predictive discrimination.

**Results:**

Of 85 identified patients, 63 were included in patient-level analyses and 446 teeth in tooth-level analyses. GR was present in 74.6% of patients. Increasing age was associated with GR (odds ratio, 1.098; *p* < 0.01), whereas sex, smoking, and Angle Class were not. At tooth level, greater wear-facet involvement was associated with both higher recession scores and greater recession depth (both *p* < 0.0001), and age was also associated with both recession outcomes. The exploratory ML-based risk model showed moderate discrimination (AUC, 0.734 ± 0.061).

**Conclusions:**

Among recession-affected teeth, greater regional wear-facet involvement was associated with greater GR severity.

**Clinical Relevance:**

The spatial distribution of wear facets may provide additional contextual information when assessing teeth with gingival recession, as greater regional involvement was associated with greater recession severity.

**Supplementary Information:**

The online version contains supplementary material available at https://doi.org/10.1007/s00784-026-07204-z.

## Introduction

Gingival recession (GR) is defined as the apical displacement of the gingival margin relative to the cemento-enamel junction, resulting in root-surface exposure [1]. It is a common clinical finding associated with dentinal hypersensitivity, root caries, non-carious cervical lesions, esthetic concerns, and impaired oral health-related quality of life [2]. A recent meta-analysis of 15 population-based studies including 37,460 participants reported a pooled global prevalence of 78.16%, increasing to 84.92% at a threshold of ≥ 1 mm, although substantial heterogeneity in case definitions and assessment methods was noted [3]. Untreated buccal recessions may also progress over time [4]. The etiology of GR is multifactorial and involves anatomical, periodontal, behavioral, and functional influences. Local susceptibility has been linked to gingival phenotype, keratinized tissue width, tooth position, and buccal bone morphology, while recession defects must also be interpreted in relation to the surrounding periodontal condition [1, 2, 5]. The contribution of occlusion and mechanical loading remains controversial. Current evidence does not support a direct causal relationship between occlusal trauma and GR, and neither premature contacts nor wear facets alone are sufficient to establish such a mechanism [6, 7]. Nevertheless, more recent clinical and experimental studies have discussed functional and mechanical factors as potential contributors in the presence of local anatomical or periodontal susceptibility [8–10]. Tooth position and local morphology are therefore of particular interest. Systematic evidence suggests that proclined or malpositioned teeth may show a higher occurrence of recession, although the overall evidence remains limited and heterogeneous [11–13]. Digital intraoral scans are particularly suited to investigate such relationships because they allow alignment, visible crown angulation, occlusal wear, and gingival margins to be assessed within the same three-dimensional dataset. Conventional clinical recession measurements are limited by access, probe angulation, examiner dependence, and millimeter resolution [14, 15]. Digital measurements on virtual models have shown improved reproducibility and lower measurement variability, while also permitting repeated assessment of identical sites and simultaneous evaluation of soft-tissue contours and dental morphology [14–16].

Archived full-arch intraoral scans may therefore provide a valuable opportunity to investigate GR together with cumulative wear patterns and other scan-visible morphological features within the same dentition. However, little is known about how scan-visible wear patterns, alignment, and crown angulation relate to recession when analyzed together. Therefore, the aim of this retrospective exploratory study was to characterize GR at patient and tooth levels and to evaluate its associations with occlusal wear facets, tooth type, alignment irregularities, and crown angulation using pseudonymized digital intraoral scans.

## Materials and methods

### Study design and setting

This retrospective exploratory observational study analyzed pre-existing full-arch digital intraoral scans obtained during routine clinical care at the University Clinic of Dentistry, Medical University of Vienna, Austria.

For each included patient, the archived digital dataset comprised maxillary and mandibular full-arch scans together with the corresponding digital interocclusal bite registration, allowing assessment of both arches in their recorded occlusal relationship. Analyses were conducted at the patient and tooth levels. All scan-based assessments were performed by one examiner (T.M.). To assess intra-examiner reliability for gingival recession depth measurements, blinded repeat measurements were performed on a representative subsample of 47 teeth (10.5% of the tooth-level sample), sampled across tooth types and patients. Reliability was quantified using the intraclass correlation coefficient (ICC (1,3); two-way mixed-effects model, absolute agreement, single measures). Patient-level analyses assessed the prevalence of gingival recession and associated patient characteristics, whereas tooth-level analyses examined recession severity in relation to wear-facet involvement, tooth type, alignment, and crown angulation.

Eligible scans were retrieved from the institutional archive for the period from 1st January 2023 to 31st July 2025. The study was approved by the Ethics Committee of the Medical University of Vienna (approval no. 1747/2025). All scans were analyzed in pseudonymized form using secured institutional systems. All measurements were performed on the archived digital models using 3D Slicer 5.10.0. The scans had been acquired using Primescan (Dentsply Sirona, Germany) and Trios 4 (3Shape, Denmark).

Linked patient information, including age at scanning, sex, and smoking status, was retrieved from the institutional electronic patient record using a pseudonymized study code. Only one scan per patient was included. If multiple eligible scans were available, the most recent scan obtained before the predefined cut-off date was selected; if this scan did not meet the quality criteria, the next most recent eligible scan was used.

### Study population and eligibility criteria

Adult patients aged ≥ 18 years with an assessable full-arch intraoral scan and assessable erupted teeth in the anterior and posterior regions were eligible. To reduce major treatment- or disease-related confounding, patients with documented previous orthodontic treatment or relevant periodontal destruction were excluded. Additional exclusion criteria were incomplete or corrupted scan files, insufficient scan quality, non-assessable soft-tissue or tooth morphology, and documented systemic conditions considered relevant to periodontal health.

**Fig. 1:**
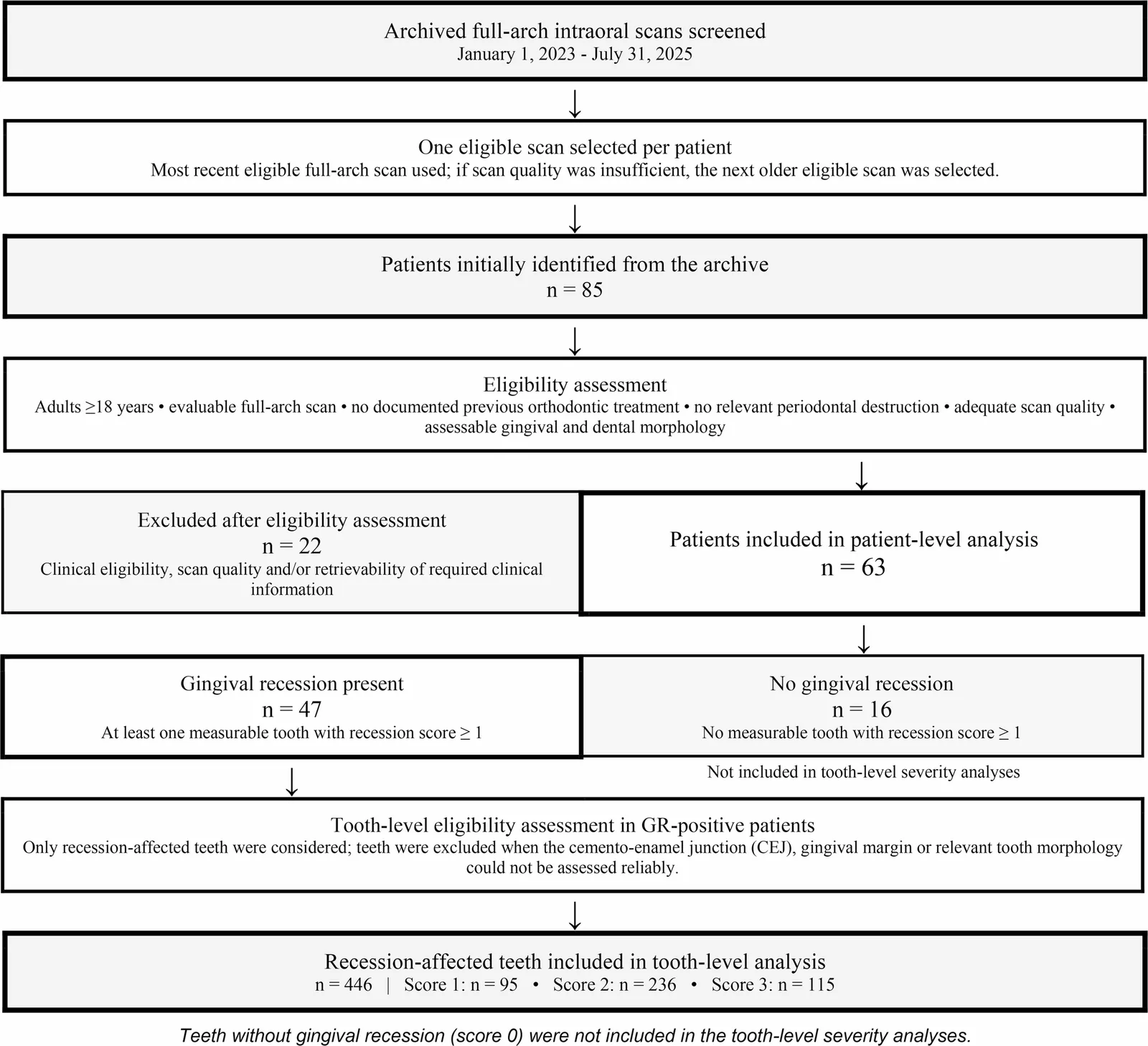
Flowchart of patient and tooth selection for the patient-level and tooth-level analyses

Because of the retrospective design, background information was not uniformly available for all patients, and no assumptions were made when variables were incompletely documented. If only individual teeth were non-assessable, these teeth were excluded from tooth-level analyses while the patient remained in the patient-level sample. The patient- and tooth-level selection process is summarized in Fig. 1.

### Digital intraoral scans and image assessment

**Fig. 2:**
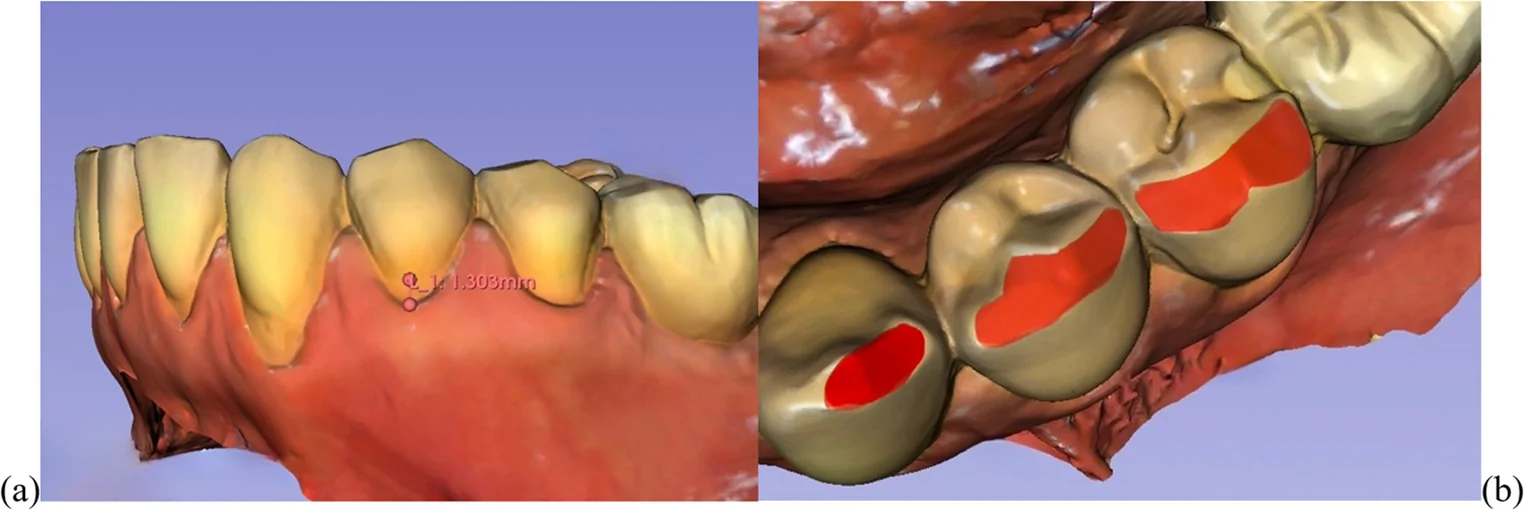
Representative scan-based assessment of gingival recession and occlusal wear. **a** Measurement of mid-buccal recession depth from the cemento-enamel junction to the gingival margin on the digital model. **b** Recording of the location and regional involvement of visible occlusal wear facets on posterior teeth

All recession- and morphology-related variables were assessed directly on the archived full-arch digital models. The three-dimensional environment allowed repeated inspection of identical sites and simultaneous evaluation of gingival contours, tooth morphology, and wear-related features. A representative example of the measurement procedure is shown in Fig. 2.

Right- and left-sided Angle Class were assessed from the maxillary and mandibular digital scans using the corresponding digital interocclusal bite registration. 

### Gingival recession assessment

Gingival recession was assessed at the mid-buccal site of each tooth. Based on the continuously measured recession depth, a predefined ordinal categorization was applied for the present analysis: 0 = no recession, 1 = recession < 1.0 mm, 2 = recession ≥ 1.0 mm and < 2.0 mm, and 3 = recession ≥ 2.0 mm. The site-specific assessment approach was informed by previous studies evaluating mid-buccal recession [8, 10]. At the mid-buccal site, the measurement points were placed at the visible cemento-enamel junction (CEJ) and the corresponding gingival margin. In addition, at the mid-buccal site, recession depth was measured as the direct three-dimensional linear distance from the cemento-enamel junction (CEJ) to the gingival margin using the digital measurement tool in 3D Slicer [15–17].

At patient level, gingival recession was defined as the presence of at least one measurable tooth with a recession score ≥ 1. Teeth without gingival recession (score 0) were not part of the tooth-level analyses. Teeth were considered non-assessable when scan artefacts or cervical morphological alterations prevented reliable identification of the CEJ or gingival margin and were excluded from the corresponding tooth-level analysis. Cervical wear, non-carious cervical lesions, restorations, or scan artefacts did not automatically lead to exclusion; teeth were considered non-assessable when these conditions prevented reliable identification of the CEJ or gingival margin. The mid-buccal site was selected because it is clinically relevant and can be assessed reliably on digital scans. Mid-buccal recession was assessed in accordance with previous site-specific studies [2, 8].

### Occlusal wear facets and derived involvement/location score

Wear-facet severity was scored using an adapted Prasad index: 0 = no facet, 1 = enamel involvement only, 2 = shallow dentin exposure, and 3 = deep dentin exposure [10]. Wear facets were included as scan-visible structural indicators of tooth wear and were not interpreted as direct proof of occlusal trauma [7].

The exact location of each wear facet was additionally recorded. This step was taken to improve comparability across different tooth classes.

Because the number of potentially involved regions differs between tooth classes, the original location data were condensed into a three-level wear-facet involvement/location score for inferential analyses:

#### Score 0

no visible wear facet.

#### Anterior teeth (incisors, canines)

score 1 = wear limited to the incisal edge; score 2 = incisal wear with at least one additional surface region.

#### Premolars

score 1 = one cusp region involved; score 2 = both cusp regions involved.

#### Molars

score 1 = one or two cusp regions involved; score 2 = three or more cusp regions involved.

The original location categories were retained for descriptive reporting, whereas the derived score was used in the main tooth-level models.

### Assessment of crown angulation and alignment

Directional crown angulation was assessed visually on the three-dimensional model and classified as no apparent angulation, mesial, distal, buccal, palatal/lingual, or a combined direction such as mesio-buccal or disto-palatal. Teeth that could not be assessed reliably were coded as not determinable. For inferential analyses, crown angulation was additionally dichotomized as no apparent angulation versus any visible angulation. The assessments were performed by T.M., a dentist who had received curricular training in orthodontics during dental education. The study was supervised by X.R.-F., an orthodontic specialist. Crown angulation and alignment were assessed by visual inspection of the archived digital models using the categorical classifications described above, and the same assessment approach was applied throughout the dataset. Alignment was categorized as normal, crowded, rotated, or spaced. Tooth position was recorded using Fédération Dentaire Internationale (FDI) notation and grouped by tooth type as incisor, canine, premolar, or molar. Broader positional groupings were used where required for descriptive analysis. The inclusion of crown angulation and alignment variables was methodologically justified because the literature suggests that tooth position, malposition, and local morphologic profile may be relevant in recession research [2]. However, in the present study these variables were included as exploratory scan-visible morphological characteristics. These variables were assessed categorically to permit retrospective evaluation across the archived digital models and were not intended to represent a quantitative orthodontic analysis or direct surrogates of a specific causal mechanism.

### Statistical analysis

Given the retrospective and exploratory archive-based design, no formal a priori sample-size or power calculation was performed. The sample size was determined by the number of eligible scan datasets available within the predefined study period. Analyses were performed at both the patient and tooth level. Descriptive statistics were used to summarize the distribution of all recorded variables. At the patient level, gingival recession was treated as a dichotomous outcome. Differences in age between groups were assessed using Welch’s t-test. Associations between categorical patient-level variables and gingival recession were examined using Chi-square or Fisher–Freeman–Halton exact tests, if 20% of the cell counts were below 5. In addition, a multivarible logistic regression analysis was performed to explore potential patient-level predictors of gingival recession. Due to the small number of observations in Angle Class III, Angle Classes II and III were combined for the logistic regression analysis to improve model stability and ensure convergence.

At tooth level, descriptive analyses included the distribution of recession scores, recession depth, tooth type, wear-facet severity, the derived wear-facet involvement/location score and patient age. Because multiple teeth were contributed by the same patient, a gamma-log generalized linear mixed model (GLMM) was used to account for clustering within patients with the recession depth in millimeters as outcome. Gamma-log was used as the data showed a right-skewed tendency. For the ordinal recession score, a cumulative link mixed model (CLMM) with a logit link function was fitted. In both models, patient was included as a random intercept.

The main fixed effects in the tooth-level analyses were the derived wear-facet involvement/location score, tooth type, alignment, crown angulation, and patient age in simplified form where applicable. Interaction effects were not included in the final models because the small sample sizes within several interaction cells would have resulted in sparse data, unstable parameter estimates, and limited statistical power. Post hoc pairwise comparisons were carried out for selected significant effects using multiplicity-adjusted testing using Tukey’s HSD. Additional exploratory associations between categorical variables were examined using Fisher–Freeman–Halton exact tests, including analyses of the relationship between wear-facet involvement/location score and alignment or crown angulation categories. A two-sided significance level of *p* < 0.05 was applied throughout.

As a supplementary proof-of-concept approach, an exploratory Random Forest risk-prediction model was used to classify higher-severity recession (≥ 2.0 mm) versus recession depths < 2.0 mm. Predictors included wear-facet involvement/location, tooth type, alignment, crown angulation, and patient age. The model used 1,000 estimators and a fixed seed for reproducibility. The analysis was repeated across 100 training/test splits, with splitting performed at the patient level to avoid data leakage. Model discrimination was assessed using the AUC, and variable contributions were explored using SHAP.

## Results

### Patient-level findings

Of 85 patients initially identified from the archive, 63 fulfilled the eligibility criteria and were included in the final patient-level analysis. Gingival recession was present in 47 patients (74.6%), and wear facets were present in 61 patients (96.8%) (Table 1).

Patients with gingival recession were older than those without gingival recession (52.29 ± 2.029 vs. 37.94 ± 2.209 years; *p* < 0.0001). In multivariable logistic regression, increasing age was associated with the presence of gingival recession (OR = 1.098, 95% CI 1.033–1.166; *p* < 0.01), whereas sex, smoking status, and right- or left-sided Angle Class were not significantly associated with gingival recession (Table 2).

**Table 1:** Percentages refer to the final patient-level analytical sample (*n* = 63)

Variable	*n* (%)
Total available patients	85
Valid cases for patient-level analysis	63
Gingival recession present	47 (74.6)
Gingival recession absent	16 (25.4)
Wear facets present	61 (96.8)
Wear facets absent	2 (3.2)
Female sex	36 (57.1)
Male sex	27 (42.9)
Smoking: yes	21 (33.3)
Smoking: no	42 (66.7)
Angle Class (right): I	32 (50.8)
Angle Class (right): II	30 (47.6)
Angle Class (right): III	1 (1.6)
Angle Class (left): I	37 (58.7)
Angle Class (left): II	23 (36.5)
Angle Class (left): III	3 (4.8)

**Table 2:** Patient-level associations with gingival recession. Age was compared using Welch’s t-test. Associations between categorical variables and gingival recession were assessed using chi-square or Fisher–Freeman–Halton exact tests, as appropriate. Multivariable logistic regression included age, sex, smoking status, and right- and left-sided Angle Class. Sparse Angle Class categories were combined for regression analysis to improve model stability

**Variable**	Univariate analysis	Multivariable logistic regression
Age	37.94 ± 2.209 years (no GR) vs. 52.29 ± 2.029 years (GR); p < 0.0001	OR = 1.098 (95% CI 1.033–1.166); p < 0.01
Sex	*p* = 0.616	*p* = 0.939
Smoking status	*p* = 0.306	*p* = 0.587
Angle Class (right)	*p* = 0.832	*p* = 0.764
Angle Class (left)	*p* = 0.466	*p* = 0.669

Abbreviations *GR* gingival recession, *OR* odds ratio, *CI* confidence interval

### Tooth-level descriptive findings

For tooth-level severity analyses, only recession-affected teeth (recession scores 1–3) were included. Teeth without gingival recession (score 0) were not included because these analyses were designed to investigate variation in recession severity among affected teeth. A total of 446 teeth were included in the tooth-level analysis, with a mean recession depth of 1.64 ± 0.82 mm. Recession score 2 was the most frequent category (52.9%), and enamel-limited wear was the most common wear grade (54.5%) (Table 3). Most teeth showed normal alignment (73.8%), while 11.9% were crowded, 9.0% rotated, and 5.4% spaced; no apparent crown angulation was recorded in 80.9% of teeth, whereas 19.1% showed visible crown angulation. Canines showed the highest mean recession depth (1.78 mm) and the highest proportion of score-3 recession (37.5%), followed by incisors, whereas premolars and molars showed lower mean recession depths.

Intra-examiner reliability for recession-depth measurements, assessed in 47 repeated tooth-level measurements, was excellent (ICC (1,3) = 0.999; 95% CI 0.997–0.999), with a mean difference of − 0.0008 ± 0.04 mm between repeated measurements.

**Table 3:** Descriptive tooth-level characteristics of the analyzed teeth. Note: Percentages refer to the 446 teeth included in the tooth-level analysis

Variable	*n* (%) or mean ± SD
Total teeth included	446
Recession depth, mm	1.64 ± 0.82
Tooth type: incisor	113 (25.3)
Tooth type: canine	88 (19.7)
Tooth type: premolar	143 (32.1)
Tooth type: molar	102 (22.9)
Recession score 1	95 (21.3)
Recession score 2	236 (52.9)
Recession score 3	115 (25.8)
Wear facet: none	66 (14.8)
Wear facet: enamel	243 (54.5)
Wear facet: shallow dentin	119 (26.7)
Wear facet: deep dentin	18 (4.0)
Alignment: normal	329 (73.8)
Alignment: crowded	53 (11.9)
Alignment: rotated	40 (9.0)
Alignment: spaced	24 (5.4)
Binary crown angulation: no apparent angulation	361 (80.9)
Binary crown angulation: any visible angulation	85 (19.1)

Abbreviations *SD* standard deviation

### Tooth-level mixed-model findings

To evaluate tooth-level associations while accounting for clustering of teeth within patients, mixed-effects models were fitted using gingival recession as both an ordinal and a continuous outcome. In the CLMM for recession score, wear-facet involvement/location score (*p* < 0.0001), tooth type (*p* = 0.010), and age (*p* = 0.022) were significantly associated with recession severity. Each additional year of age was associated with higher odds of being in a higher recession-score category (OR = 1.030, 95% CI 1.005–1.057). Alignment (*p* = 0.627) and binary crown angulation (*p* = 0.641) were not significantly associated with recession score.

In the gamma-log GLMM for recession depth, wear-facet involvement/location score (*p* < 0.0001) and age (*p* = 0.013) were significantly associated with recession depth. Each additional year of age was associated with a mean ratio of 1.007 (95% CI 1.002–1.012) for recession depth. Alignment (*p* = 0.084) and tooth type (*p* = 0.0501) did not reach statistical significance, whereas binary crown angulation was not significantly associated with recession depth (*p* = 0.254). 

**Fig. 3:**
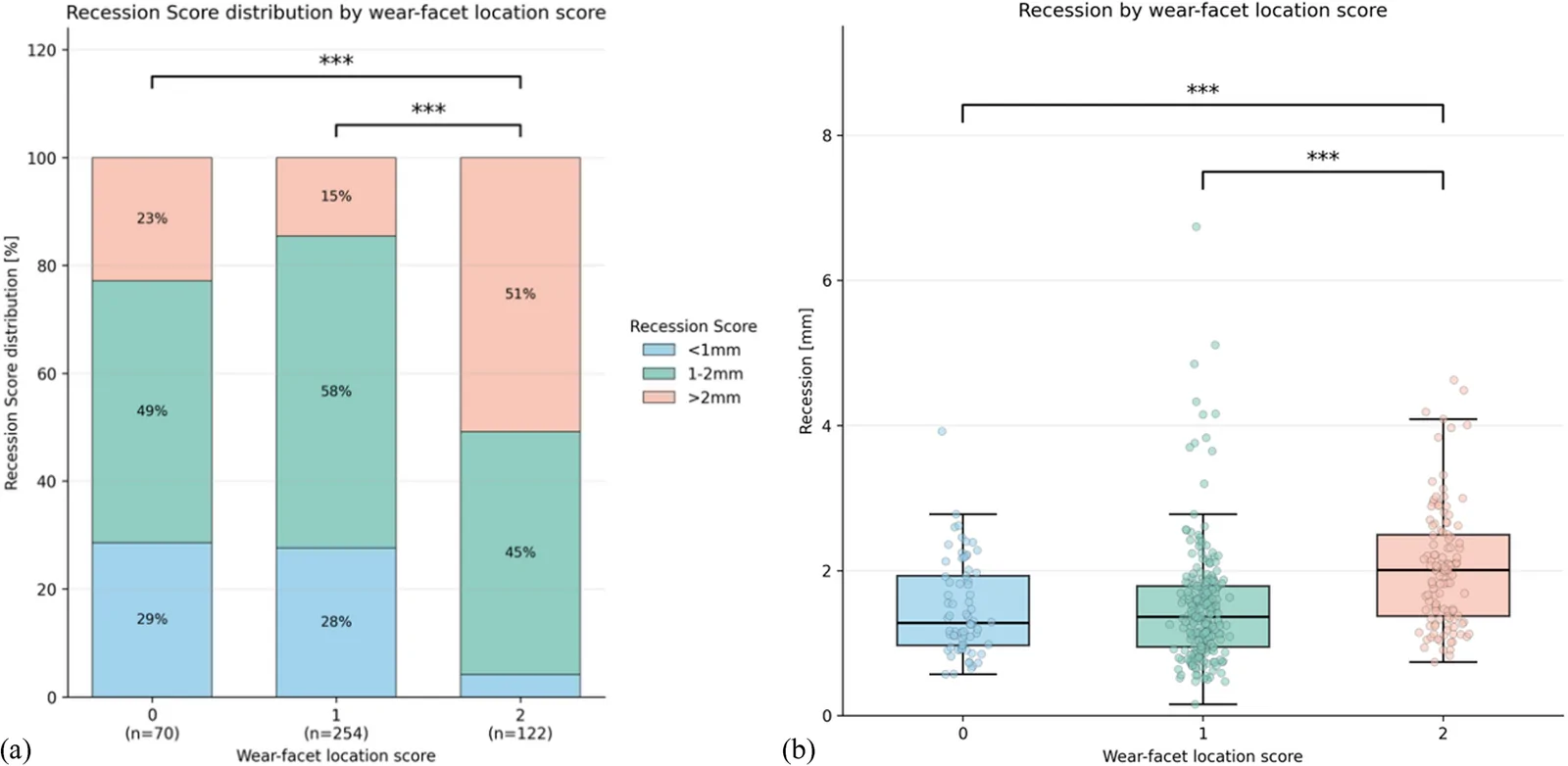
Tooth-level gingival recession according to wear-facet involvement/location score. (**a**) Recession score distribution by wear-facet involvement/location score. (**b**) Recession depth (mm) by wear-facet involvement/location score. Higher wear-facet involvement/location scores were associated with more severe gingival recession

Tukey-adjusted post hoc comparisons for the CLMM showed that wear-facet involvement/location score 2 differed significantly from scores 0 and 1 (both *p* < 0.0001), whereas scores 0 and 1 did not differ (*p* = 0.432). Significant tooth-type differences were observed between incisors and canines (*p* = 0.020) and between canines and premolars (*p* = 0.022). For the GLMM recession depth, score 2 also differed significantly from scores 0 and 1 (both *p* < 0.0001), whereas scores 0 and 1 did not differ (*p* = 0.749) (Fig. 3). A more comprehensive summary of the results of the final age-adjusted mixed-effects models, including contrast-specific p-values, effect estimates (GLMM: mean ratios; CLMM: odds ratios), and corresponding 95% CI, are provided in Supplementary Tables S1 and S2. 

### Exploratory categorical associations

Exploratory categorical analyses showed that the derived wear-facet involvement/location score was significantly associated with alignment category in the Fisher–Freeman–Halton exact test (*p* = 0.005). By contrast, no significant association was observed between the wear-facet involvement/location score and binary crown angulation (*p* = 0.209). These findings suggest that the distribution of wear-facet involvement may vary across alignment categories, whereas no corresponding categorical relationship was evident for crown angulation. The calculated adjusted standardized residuals showed residuals larger than 1.96 at wear-facet involvement/location score 0 for normal angulation and for location score 1 for crowded, while smaller residual values than − 1.96 were found for location score 1 for normal angulation and for a location score 0 in crowded. A residual value larger than 1.96 indicates higher frequency than expected while values smaller than − 1.96 a lower frequency than expected [18].

### Exploratory predictive modelling

**Fig. 4:**
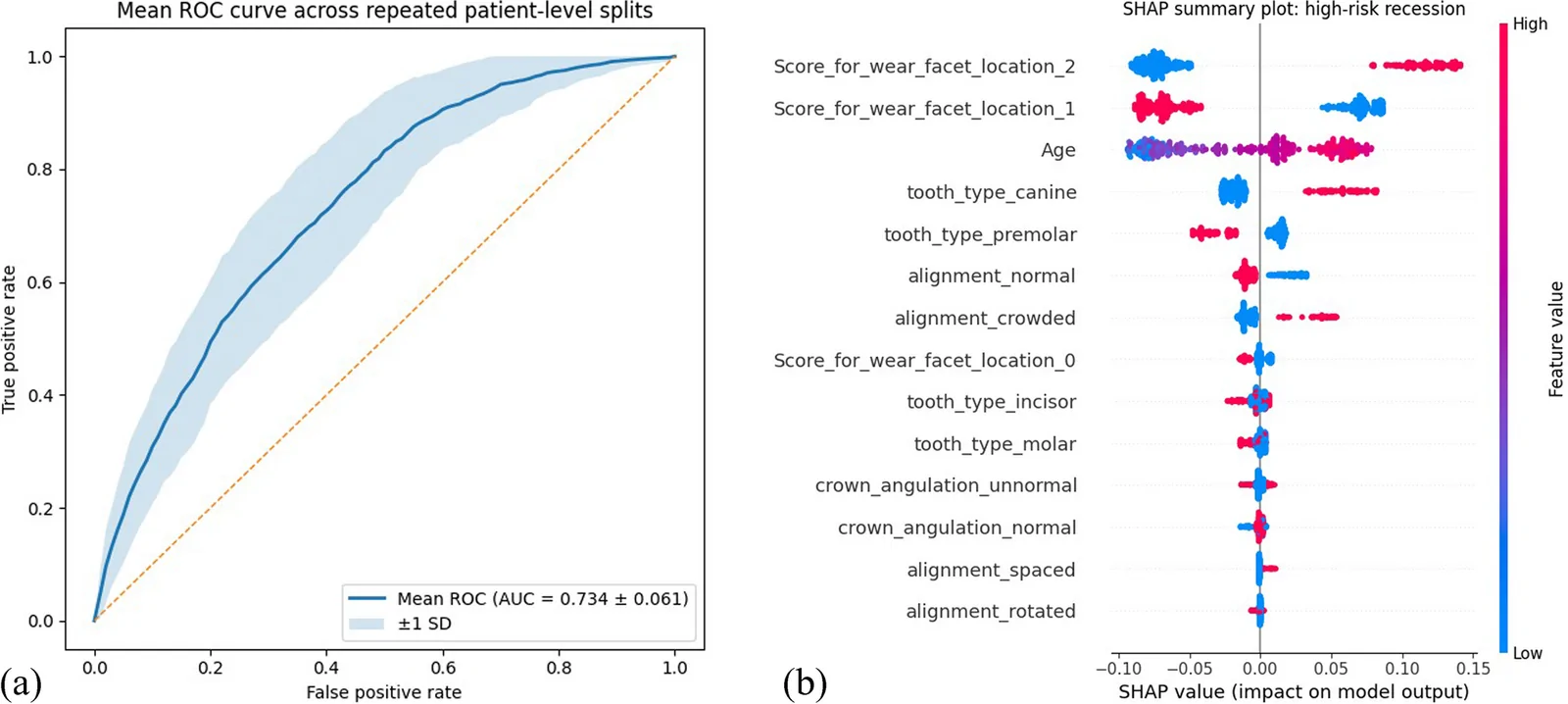
Exploratory machine-learning risk model for high-risk gingival recession. **a** Receiver operating characteristic (ROC) curve across repeated patient-level splits. The mean area under the curve (AUC) was 0.734 ± 0.061, indicating moderate discriminatory performance. **b** SHAP summary plot showing the relative importance and direction of the evaluated model variables. Wear-facet involvement/location, age, and tooth type showed the greatest contributions to model output

As a supplementary proof-of-concept analysis, an exploratory ML-based risk model was developed to complement the primary mixed-effects analyses. The model showed moderate discrimination across repeated patient-level splits (AUC 0.734 ± 0.061). SHAP analysis identified wear-facet involvement/location as the most influential feature, followed by age and tooth type, whereas alignment and crown angulation contributed less. Higher wear-facet involvement, greater age, and canine tooth type tended to shift model predictions toward the high-risk recession class (Fig. 4). 

## Discussion

This exploratory retrospective scan-based study aimed to investigate associations between gingival recession severity, occlusal wear, and scan-visible dental morphology at patient and tooth levels. Mixed-effects models identified the derived wear-facet involvement/location score as the most consistent tooth-level correlate of recession severity, with increasing regional involvement associated with both higher ordinal scores and greater recession depth. Tooth type contributed to the ordinal model, canines showed the highest descriptive recession values, and age was the only patient-level variable significantly associated with gingival recession. Alignment and visible crown angulation showed no stable independent associations. These findings suggest that the spatial distribution of wear may provide information beyond its mere presence.

The distinction between the presence, severity, and spatial distribution of wear is central to these findings. Tooth wear accumulates after eruption through the interaction of function, aging, and individual behavioral or chemical exposures. Longitudinal digital studies have shown measurable progression, marked interindividual variation, and non-uniform involvement of load-bearing regions; posterior wear also differs by tooth type and jaw over longer observation periods [19–21]. These observations suggest that the regional distribution of wear may provide descriptive information that is not captured by a single whole-tooth presence or severity category.

This may explain why the refined score was more informative than a binary wear variable. Wear was highly prevalent in the present sample, limiting the discriminatory value of presence-versus-absence recording. Al-Seelawi et al. similarly detected wear in all examined young adults and showed that digital models identified early changes in areas that were difficult to inspect clinically, while agreement between conventional and digital scoring was only moderate [21]. A conventional clinical index may therefore not capture all spatial information available in a three-dimensional model. The derived score used here was designed to incorporate this regional information by recording the extent of involvement across tooth-specific surfaces. However, wear facets cannot establish excessive occlusal loading, bruxism, or trauma from occlusion. Fan and Caton described them as surrogate indicators that may arise from physiological contacts, parafunction, or other processes [7]. Previous clinical studies have consequently produced conflicting findings: Prasad et al. and studies in younger patients reported associations between recession, wear, and excursive interferences, whereas Kundapur et al. found no significant relationship with the simple presence of facets or fremitus [10, 22, 23]. Rather than supporting either a direct causal theory or complete functional irrelevance, this heterogeneity may partly reflect differences in study populations, definitions, and methods of morphological assessment.

The present findings are therefore best interpreted within a multifactorial framework. In the broader literature, tooth wear and gingival recession have been associated with multiple anatomical, periodontal, behavioral, chemical, and potentially functional factors. These factors were not comprehensively assessed in the present study and are therefore considered here as contextual explanations rather than mechanisms demonstrated by the present data. Consequently, their cross-sectional association may reflect shared exposures or susceptibility rather than a direct mechanistic relationship. Mascardo et al. found that midfacial recession was associated with reduced keratinized tissue width, thinner gingiva, buccal bone dehiscence, and the buccolingual three-dimensional profile [2]. Surface morphology therefore represents only one component of a broader dento-periodontal system. Because several relevant clinical and functional variables were unavailable in the present retrospective dataset, the mechanisms underlying the observed association cannot be determined from the scan data alone.

This interpretation also provides context for the tooth-type finding. Canines showed the highest mean recession depth and the greatest proportion of severe scores, while tooth type remained significant in the ordinal model. Previous studies have demonstrated tooth- and arch-specific differences in gingival recession distribution and gingival dimensions, supporting the relevance of regional anatomical characteristics when interpreting recession patterns [24, 25]. Tooth type should therefore be regarded as a descriptive marker encompassing several tooth-specific anatomical and positional characteristics rather than as an isolated causal factor. By contrast, alignment and visible crown angulation were not independently associated with recession in the present study. In an untreated control population, greater dental crowding was associated with a higher number of gingival recession sites [26]. These findings suggest that tooth position may be relevant in some populations, although the present study did not demonstrate an independent association for alignment or visible crown angulation.

The association with age is consistent with the cumulative nature of recession and tooth wear. Wear progresses over time, and untreated recession defects may enlarge or new defects may develop [4, 20]. Age should not be interpreted as a direct causal factor; rather, it may reflect cumulative exposure to multiple anatomical, periodontal, inflammatory, behavioral, chemical, and functional influences. The present archive-based sample was selected to reduce major documented treatment- and disease-related confounding; however, functional status was not prospectively assessed. Consequently, undiagnosed parafunction, brushing behavior, diet, and other lifetime exposures cannot be excluded or disentangled in the present dataset [4, 27].

A major strength of this study is the simultaneous assessment of gingival margins, wear distribution, alignment, crown angulation, and tooth type within the same three-dimensional dataset. Digital models permit repeated site inspection and may reduce some limitations of conventional probe measurements, including restricted access, angulation, millimeter resolution, and examiner variability [27]. At the same time, digital workflows remain heterogeneous, and measurement definitions, software procedures, and the newly derived score require standardization and external validation [16].

The exploratory ML model showed moderate discrimination and identified wear-facet involvement/location, age, and tooth type as the variables with the greatest contributions to model output. Previous predictive models have mainly addressed treatment-related gingival recession or broader periodontal outcomes, whereas the present proof-of-concept model focuses on scan-visible wear and morphology [28]. The broadly consistent pattern across the ML and mixed-effects analyses supports the internal consistency of the findings and provides a basis for future scan-based recession-risk modelling. However, the model remains exploratory and requires external validation before clinical application.

Although this study provides new insights into the spatial assessment of occlusal wear in relation to gingival recession, several limitations should be considered. The retrospective cross-sectional design precludes causal or temporal conclusions. Important clinical covariates, including periodontal phenotype, keratinized tissue width, buccal bone morphology, brushing behavior, diet, bruxism, and detailed functional parameters, were unavailable or incompletely documented. Because the study relied on archived scans and clinical records, eligibility assessment depended on record completeness and scan assessability. Identification of the CEJ on digital models may be challenging in the presence of cervical alterations or scan artefacts; although non-assessable teeth were excluded, the exact number excluded specifically because of uncertain CEJ identification could not be reliably reconstructed. All scan-based assessments were performed by the same examiner, T.M., who had received curricular training in orthodontics during dental education, to maintain consistency across the study sample. The study was supervised by X.R.-F., an orthodontic specialist. Intra-examiner reliability for recession-depth measurements was excellent; however, no separate repeatability assessment was performed for the exploratory categorical assessments of crown angulation and alignment. These variables were assessed by visual inspection of the archived digital models and orthodontic clinical judgment rather than by standardized quantitative measurements. Accordingly, inter-examiner reproducibility of these categorical assessments could not be evaluated and this limitation should be considered when interpreting the corresponding exploratory analyses. Finally, the modest sample size limited the assessment of more complex model effects, and the exploratory machine-learning model requires validation in larger independent datasets [2, 7, 16].

## Conclusion

The spatial distribution of occlusal wear was associated with gingival recession severity among recession-affected teeth, with greater regional wear-facet involvement associated with greater recession severity. However, these cross-sectional findings do not establish a causal or functional relationship. Further prospective studies are needed to evaluate the clinical relevance of this association.

## Supplementary Information

Below is the link to the electronic supplementary material.

Supplementary Material 1 (DOCX 25.9 KB)

## Data Availability

The data analyzed during the current study are not publicly available due to data protection and privacy requirements. Access may be granted upon reasonable request to the corresponding author and with permission of the Medical University of Vienna, subject to applicable institutional, data protection, and ethical requirements; additional regulatory approvals and a data-use agreement are required.

## Declarations

### Use of generative artificial intelligence

During manuscript preparation, ChatGPT (OpenAI) was used for language editing and improvement of readability. All AI-assisted suggestions were critically reviewed and revised by the authors, who take full responsibility for the final manuscript.
