# Supplementary material for: Retrospective 3D analysis of gingival recession and occlusal morphology based on digital intraoral scans: an exploratory study
Source: Clin Oral Investig. 2026 Oct 9;30(11):515. doi: 10.1007/s00784-026-07204-z (PMC13652946; doi:10.1007/s00784-026-07204-z)
Supplement: Supplementary file 1 [file 784_2026_7204_MOESM1_ESM.docx]

| **Contrast** | **p-value** | **Mean ratio** | **95%-CI** |
| --- | --- | --- | --- |
| Alignment |  |  |  |
| Crowded / normal | 0.073 | 1.191 | [0.989; 1.434] |
| Rotated / normal | 0.989 | 1.022 | [0.857; 1.219] |
| Rotated / crowded | 0.264 | 0.858 | [0.691; 1.065] |
| Spaced / normal | 0.766 | 1.086 | [0.874; 1.349] |
| Spaced / crowded | 0.815 | 0.912 | [0.700; 1.194] |
| Spaced / rotated | 0.933 | 1.062 | [0.819; 1.377] |
|  |  |  |  |
| Crown angulation |  |  |  |
| Any visible angulation / no apparent angulation | 0.254 | 0.941 | [0.848; 1.044] |
|  |  |  |  |
| Tooth type |  |  |  |
| Canine / incisor | 0.091 | 1.137 | [0.987; 1.309] |
| Premolar / incisor | 0.991 | 1.016 | [0.886; 1.165] |
| Premolar / canine | 0.127 | 0.894 | [0.783; 1.020] |
| Molar / incisor | 1.000 | 0.997 | [0.854; 1.163] |
| Molar / canine | 0.106 | 0.877 | [0.756; 1.018] |
| Molar / premolar | 0.982 | 0.981 | [0.863; 1.116] |
|  |  |  |  |
| Location Score |  |  |  |
| 1 / 0 | 0.749 | 0.961 | [0.843; 1.094] |
| 2 / 0 | <0.0001 | 1.299 | [1.128; 1.496] |
| 2 / 1 | <0.0001 | 1.352 | [1.221; 1.500] |
|  |  |  |  |
| Age | 0.013 | 1.007 | [1.002; 1.012] |

**Supplementary Material**

**Retrospective 3D Analysis of Gingival Recession and Occlusal Morphology Based on Digital Intraoral Scans: An Exploratory Study**

Supplementary Table S1: Contrast-specific p-values, mean ratios, and 95% confidence intervals from the final age-adjusted gamma-log generalized linear mixed model (GLMM).

Model estimates for CLMM:

| **Contrast** | **p-value** | **Odds ratio** | **95%-CI** |
| --- | --- | --- | --- |
| Alignment |  |  |  |
| Crowded / normal | 0.784 | 1.459 | [0.519; 4.104] |
| Rotated / normal | 0.984 | 1.150 | [0.429; 3.081] |
| Rotated / crowded | 0.958 | 0.788 | [0.234; 2.654] |
| Spaced / normal | 0.744 | 1.654 | [0.460; 5.947] |
| Spaced / crowded | 0.997 | 1.134 | [0.237; 5.425] |
| Spaced / rotated | 0.924 | 1.438 | [0.322; 6.430] |
|  |  |  |  |
| Crown angulation |  |  |  |
| Any visible angulation / no apparent angulation | 0.641 | 0.869 | [0.481; 1.570] |
| Tooth type |  |  |  |
| Canine / incisor | 0.020 | 2.535 | [1.112; 5.780] |
| Premolar / incisor | 0.988 | 1.102 | [0.512; 2.374] |
| Premolar / canine | 0.022 | 0.435 | [0.206; 0.920] |
| Molar / incisor | 0.982 | 1.136 | [0.478; 2.696] |
| Molar / canine | 0.069 | 0.448 | [0.193; 1.042] |
| Molar / premolar | 1.000 | 1.030 | [0.503; 2.110] |
|  |  |  |  |
| Location Score |  |  |  |
| 1 / 0 | 0.432 | 0.679 | [0.326; 1.415] |
| 2 / 0 | <0.0001 | 4.871 | [2.098; 11.310] |
| 2 / 1 | <0.0001 | 7.173 | [3.826; 13.448] |
|  |  |  |  |
| Age | 0.022 | 1.030 | [1.005; 1.057] |

Supplementary Table S2: Contrast-specific p-values, odds ratios, and 95% confidence intervals from the final age-adjusted cumulative link mixed model (CLMM).
